# Maternal deaths by suicide in Queensland, Australia, 2004–2017: an analysis of maternal demographic, psychosocial and clinical characteristics

**DOI:** 10.1007/s00737-021-01107-6

**Published:** 2021-06-22

**Authors:** Caitlin Modini, Stuart Leske, Susan Roberts, Nikki Whelan, Andrea Chitakis, David Crompton, David Ellwood

**Affiliations:** 1 School of Medicine and Dentistry, Griffith University, Gold Coast Campus, G40, 1 Parklands Drive, Southport, QLD 4215 Australia; 2 Australian Institute for Suicide Research and Prevention, School of Applied Psychology, Griffith University, 176 Messines Ridge Road, Mt Gravatt, QLD M094122 Australia; 3 Lavender Mother and Baby Unit, Gold Coast University Hospital, 1 Hospital Boulevard, Southport, QLD 4215 Australia; 4 Department of Obstetrics and Gynaecology, The Wesley Hospital, 40 Chasely Street, Suite 20, Level 2, Auchenflower, QLD 4066 Australia; 5 Queensland Maternal and Perinatal Quality Council, Queensland Health, Queensland Health Quality and Safety, 15 Butterfield St, Herston, QLD 4006 Australia; 6 Translational Research Institute Australia, Neuroimaging, 37 Kent St, South Brisbane, QLD 4101 Australia; 7 Faculty of Health, Queensland University of Technology, 2 George Street, Brisbane City, QLD 4000 Australia; 8 Maternal-Fetal Medicine, Gold Coast University Hospital, Southport, QLD 4215 Australia

**Keywords:** Suicide, Peripartum, Obstetrics

## Abstract

To characterise the demographic and clinical characteristics of women who died by suicide in the perinatal period to inform and improve suicide prevention strategies. Retrospective analysis of maternal suicides during and within 1 year after the end of pregnancy in Queensland between January 2004 and December 2017. Outcomes measured included timing of death in relation to pregnancy, sociodemographic and clinical characteristics and health service use prior to death. There were 65 deaths by suicide in the study period; six occurred during pregnancy, 30 occurred after a live birth, 22 occurred after a termination of pregnancy and seven followed a miscarriage or stillbirth. Most suicides were late maternal deaths. Women were younger, and more likely to identify as Aboriginal or Torres Strait Islander, when compared to all women giving birth for the same time period. Most women had a prior mental health diagnosis, most commonly depression. Over half of women had recent relationship separation or conflict prior to death. Perinatal women had higher rates of death by violent means than all women in Queensland who died by suicide during the same time period. The demographic, psychosocial and clinical characteristics of a group of women who died by suicide have been described, and this shows a high proportion of women with a prior mental health diagnosis. To reduce maternal mortality, psychosocial screening must be implemented broadly and continued until the end of the first year postpartum. Similar screening attention is needed for women who had a termination of pregnancy, miscarriage or stillbirth.

**Supplementary Information:**

The online version contains supplementary material available at 10.1007/s00737-021-01107-6.

## Introduction

Improvements in maternal healthcare over recent decades have been vast, making Australia one of the safest countries in the world to give birth. However, as deaths due to obstetric complications decrease, a trend towards increasing psychosocial causes of maternal mortality is emerging. This trend is reflected nationally, with suicide being the second-leading cause of maternal death in Australia in 2017 and the third-leading cause of maternal death from 2008 to 2017 (Australian Institute of Health and Welfare [AIHW] 2019). In the most recent Queensland report, which also includes late maternal deaths (those occurring up until 1 year after the end of pregnancy), suicide was the second-highest cause of maternal mortality.

While pregnancy may confer a protective effect against suicide (Gissler 2005), this is likely to lessen in the early postpartum period (Howard et al. 2014). Furthermore, for some at-risk women, including those who have had a termination of pregnancy, suicide risks may be elevated early in the period after the pregnancy has ended (Gissler 2005; Khalifeh et al. 2016).

Suicide prevention is a national policy priority in Australia (Battams and Robards 2019). There is no other time in life when women are most likely to have more contact with health services than in pregnancy, so this is a unique opportunity to screen for, identify and treat women at risk for maternal suicide. A better understanding of the factors that increase the risk is crucial to inform and improve prevention strategies and ultimately reduce overall maternal mortality. Sociodemographic and psychosocial characteristics, as well as patterns of health service use of women who have died by suicide, all need consideration.

In light of this, our main aim was to review deaths by suicide of women in Queensland during pregnancy or within 1 year after the end of pregnancy. This review included assessing the different pregnancy outcomes, the timing of suicide relative to pregnancy, sociodemographic characteristics, prior mental health history and treatment and health service usage.

## Methods

The Human Research Ethics Committee (HREC) of Griffith University (GU Ref Nos: 2018/261; 2010/537) and the Victorian Department of Justice and Community Safety HREC (CF/18/12771) approved this research. Data came from two sources: the Queensland Maternal and Perinatal Quality Council (QMPQC) and the Queensland Suicide Register (QSR). The Maternal Mortality Subcommittee of the QMPQC receives mandatory notifications of maternal deaths from medical practitioners, midwives and hospitals under public health legislation. The QMPQC accesses and reviews hospital, antenatal and private clinic records. The QSR (1990–2015) monitors all deaths by suicide in Queensland residents. Data sources for the QSR include police reports of deaths to coroners, toxicology and autopsy reports and coronial findings.

Data abstracted included:

- Sociodemographic factors (maternal age, country of birth, ethnicity, marital status, weeks of gestation or number of months post-pregnancy at the time of death);
- Mental health history (documented diagnoses, current pharmacotherapy, prior suicidality, prior suicide attempts and current or previous substance use);
- Social history (living arrangements, employment status, significant life events preceding death), clinical history (preterm birth, adverse pregnancy events);
- Suicide methods and
- Toxicology results.

QMPQC records provided most pregnancy-related information, and QSR records provided most psychiatric information. We cross-checked data sources and combined into SPSS Statistics, where we generated descriptive data such as frequencies and percentages. Data on age, ethnicity and remoteness provided to the QMPQC for all women giving birth in Queensland for the same period served as a comparison group. Data on suicide methods used by all women in the same age group in Queensland during the study time period was sourced from the QSR and used for comparison.

## Results

We identified 65 maternal deaths by suicide. There were 61 maternal deaths by suicide (all-cause *N* = 286) reported to the QMPQC in the 14 years between 1 January 2004 and 31 December 2017. About half of the 61 cases reported to the QMPQC were not identified as pregnancy-related deaths in the QSR. This discrepancy is likely because these deaths occurred up until 12 months post-pregnancy, and the QSR does not capture the age of dependants or dates of childbirth. There were eight suicides coded as pregnancy related in the QSR that were verified to be maternal deaths for which the QMPQC did not receive reports. In total then, we identified 69 possible maternal suicides. We excluded four due to uncertainty around the nature of their death, retaining 65 deaths classified as a ‘probable’ suicide or a suicide ‘beyond reasonable doubt’ for analysis.

**Fig. 1:**
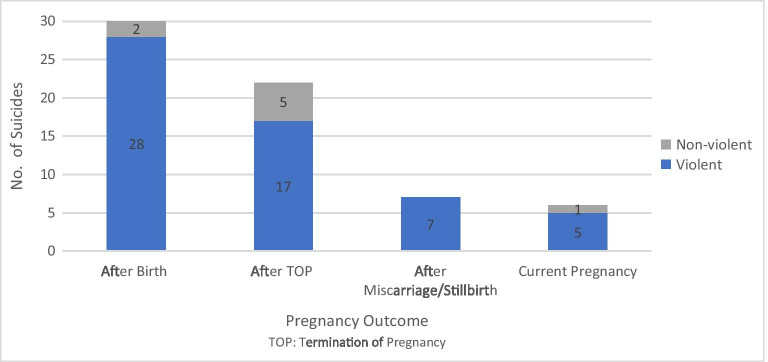
Pregnancy outcome and method of suicide

Most maternal suicides followed a live birth (*n* = 30, 46%). A third (*n* = 22, 34%) occurred up to 12 months after a termination of pregnancy (see Fig. 1). The remainder either occurred during pregnancy (*n* = 6, 9%) after a miscarriage (*n* = 6, 9%) or a stillbirth (*n* = 1, 2%). Of the women who gave birth, all but one birthed in a public hospital, while all but two of the terminations of pregnancy occurred in the private sector (see Table 1). One-third of women who gave birth had a birth complication. Only one woman who gave birth did not receive any antenatal care.

**Table 1:** Health service type and timing of the death of women who died by suicide, by pregnancy outcome

	During pregnancy (*n* = 6)	After birth (*n* = 30)	After ToP (*n* = 22)	After miscarriage (*n* = 6)	Total^a^ (*n* = 64)	
					Count (*n*)	Total %
Pregnancy care type						
Public sector	3	29	2	2	36	56%
Private sector	2	1	20	1	24	38%
N/A	1	0	0	3	4	6%
Timing of death						
During pregnancy	6	0	0	0	6	9%
0–42 days after end of pregnancy	0	5	5	1	11	17%
43–365 days after end of pregnancy	0	25	17	5	47	73%

*ToP* termination of pregnancy

^a^Stillbirth excluded from the analysis

### Timing of death

Six deaths occurred during pregnancy, and eleven (17%) occurred within 0 to 42 days after the end of pregnancy. Almost three quarters (*n* = 48, 74%) of suicides were late maternal deaths, occurring between 43 and 365 days after the end of pregnancy.

### Demographics

Women dying by suicide were on average 26 years old (range; 15–40 years old). When compared to data for all women giving birth in Queensland for this period, the proportion of women less than twenty was three times that of the overall cohort (Queensland Health 2017). As Table 2 indicates, eleven women (17%) identified as Aboriginal or Torres Strait Islander and four as Māori (6%). In comparison, Aboriginal and Torres Strait Islander women comprised only 6% of those giving birth in Queensland for this time (Queensland Health 2017). Twelve women (18%) were born outside Australia, and one of these women required a translator when attending health services. The median socio-economic index for areas (SEIFA) was four, with 48% of women living in a suburb with a SEIFA score of three or less. These areas, by definition, are the most disadvantaged 30% of neighbourhoods in Queensland. Almost half (49%) of women were living with their spouse or de facto partner at the time of their death. Twenty-nine (45%) women were unemployed at the time of death. Other demographic information such as remoteness of area was analysed and was consistent with the overall cohort of women who gave birth during the same period.

**Table 2:** Demographic characteristics of women who died by suicide with selected comparisons to the whole birthing cohort

Women who died by suicide			Whole birthing cohort
Characteristic	Count (*n*)	% of total	% of total
Age group			
15–19	10	15%	5%
20–24	22	34%	76%
25–29	11	16.9%	
30–34	13	20%	
35–39	7	11%	19%
40–44	2	3%	
Relationship status			
Married/de facto	32	49%	
Separated	5	8%	
Single	17	26%	
Widowed	1	2%	
Unknown	10	15%	
Living arrangements			
With spouse	32	49%	
With parents/friends	17	26%	
Institution	2	3%	
Alone	6	9%	
Unknown	8	12%	
Employment status			
Employed/student	16	25%	
Unemployed	29	45%	
Home duties	5	8%	
Unknown	15	23%	
Country of birth			
Australia	53	82%	
Outside Australia	12	18%	
Ethnicity			
Caucasian	39	60%	
Aboriginal/TSI	11	17%	6
Māori	4	6%	
Asian	3	5%	
Other	2	3%	
Unknown	6	9%	

*TSI* Torres Strait Islander

### Clinical characteristics

Most women (71%) who died by suicide had at least one prior mental health diagnosis (see Table 3). The most common diagnosis was depression (51%), followed by anxiety (17%) and postpartum depression (12%). Of the 33 women with depression, nine had a generalised anxiety disorder, one had a personality disorder, one had post-traumatic stress disorder and fifteen had a history of substance use. Eighteen women (28%) had more than one mental health diagnosis recorded. Nineteen (29%) women had no prior mental health diagnoses. Of the women who had documented mental health treatment, eight women had general practitioner (GP) managed care, 22 had recorded contact with outpatient mental health services and nine women had received inpatient care. Twenty-six (40%) had no recorded mental health treatment. There was documented contact with a mental health professional in the 3 months before death in 25% of the women. Of the women who died while pregnant or after giving birth, ten (28%) had contact with mental health services during pregnancy. Four (11%) women reportedly ceased medication for their mental health condition during pregnancy.

**Table 3:** Psychiatric history of women who died by suicide

Maternal suicides			All non-maternal suicides
Characteristic	Count	% of total	% of total
Alcohol consumed before suicide			
Yes	27	42%	
Suicide method			
Violent	57	88%	71%
Non-violent	8	12%	29%
Number of psychiatric diagnoses			
1 diagnosis	28	43%	
2 diagnoses	14	22%	
3/more diagnoses	4	6%	
None known	19	29%	
Diagnoses			
Depression	33	51%	
Postnatal depression	8	12%	
Bipolar disorder	3	5%	
Psychotic disorder	4	6%	
Substance use disorder	3	5%	
Generalised anxiety disorder	11	17%	
Eating disorder	1	2%	
Personality disorder	1	2%	
Post-traumatic stress disorder	1	2%	
Nil recorded	19	29%	
Highest level of mental health care			
GP care	8	12%	
Outpatient services	22	34%	
Inpatient services	9	14%	
Nil mental health care recorded	26	40%	
Mental health care in the last 3 months			
Yes	16	25%	
Medication prescribed			
Antidepressant	19	29%	
Antipsychotic	6	9%	
Benzodiazepine	9	14%	
Nil known	35	54%	
Previous suicidality			
Yes	40	62%	
Previous suicide attempt			
Yes	23	37%	
Illicit drug use			
Yes	27	42%	
History of domestic violence			
Yes	12	19%	
Recent relationship event			
Conflict	23	35%	
Separation	12	19%	
None	30	46%	

*GP* general practitioner

Antidepressants were the most commonly prescribed medication (29%) at the time of death. Many women had a history of either expressing or presenting with suicidal ideation (62%) or had previously attempted suicide (37%). However, this differed by pregnancy outcome. Forty-one percent of women who died in the late postpartum period had already attempted suicide, compared to 27% who died in the early postpartum period and 17% of women who died during pregnancy. Alcohol consumption before death and a history of illicit drug use were equally prevalent (*n* = *27*, 42%). The substances most commonly misused were cannabis (25%) and amphetamines (19%). Intimate partner violence reportedly occurred for twelve (19%) women. More than half of women (54%) had a recent relationship separation or conflict occurring before death. Twenty-nine women reportedly experienced other life events, the most common of which were bereavement (*n* = 7, 10%), family conflict (*n* = 6, 9%), child custody disputes and suicide in their social group (*n* = 4, 6% for both).

An Edinburgh Postnatal Depression Scale (EPDS) assessment was available for one woman who died during pregnancy and fourteen of thirty women who gave birth. The median score was eight. Two women scored at or above thirteen, the threshold warranting further mental health assessment (Centre of Perinatal Excellence 2019).

### Suicide methods

Hanging, strangulation or suffocation was the most common suicide method (78%). Poisoning by drugs or motor vehicle exhaust gas accounted for 12% of deaths. The remainder were other violent suicide methods, and overall, 88% of women died using violent suicide methods. In the age-matched comparison group of all women who died by suicide, hanging, strangulation and suffocation was also the most common suicide method (54%). Overall, 71% of women died by violent methods of suicide.

## Discussion

Most deaths by suicide occurred after giving birth, although over one-third of suicides followed the termination of pregnancy, and this may be a group of women requiring an increased level of care. Following the 2018 legislative changes in Queensland that decriminalised abortion, new clinical guidelines have been developed that emphasise the need for psychosocial support, screening for mental illness and further referral as required (Queensland Clinical Guidelines 2019).

While deaths occurred across the entire perinatal period, they were most common in the late maternal period (> 42 days after the end of pregnancy), and this is consistent with the existing literature (Thornton et al. 2013). This finding is important, as many screening and prevention efforts for perinatal mental illness only extend to the early postpartum period, with the last recommended screening occurring 6–12 weeks postpartum (Centre of Perinatal Excellence 2019). While many of these late maternal deaths may not be directly related to the pregnancy, they indicate the overall health of a population, allowing insight into the social and domestic factors that may make women vulnerable in the postpartum period.

Aboriginal and Torres Strait Islander women, and women living in low SEIFA areas, were overrepresented in this study. Women who died by suicide tended to be younger than the average Queensland mother, a finding that contrasts with data from the Centre for Maternal and Child Enquiries in the UK (Cantwell et al. 2011).

Mental illness frequently precedes death by suicide, and this is evident in this data (Bachmann 2018). Mood disorders such as depression and anxiety were the most common diagnoses in this study. While much of the attention towards perinatal mental health is focused on the serious but rare outcome of postpartum psychosis (Khalifeh et al. 2016), psychotic disorders in this cohort were uncommon. Depression is likely underrecognised as a serious risk factor for maternal suicide (Khalifeh et al. 2016). Furthermore, many women who had a diagnosis of either depression or postpartum depression were not receiving active treatment, consistent with UK findings (Petersen et al. 2011). Information about the relative safety of use of selective serotonin receptor inhibitor medication during pregnancy (Prady et al. 2017; Molenaar et al. 2019) should be conveyed to women as part of the decision-making process early in pregnancy or ideally in the pre-conception period, and treatment providers should be comfortable prescribing these medications to women during pregnancy. Psychological, lifestyle and social support strategies could also be discussed and regular monitoring of women ceasing medication appears necessary.

Previous suicidality and suicide attempts, well-recognised risk factors for suicide (Mościcki 1997), preceded death in many women. Women who died in the late postpartum period were more likely to have had a prior suicide attempt, compared to women who died sooner after the end of pregnancy or while pregnant.

As seen in this cohort, there is often a range of other psychosocial stressors in the lives of women who die by suicide in the perinatal period, such as intimate partner violence and drug and alcohol misuse (Esscher et al. 2016). In this study, relationship conflict or breakdown frequently occurred before suicides. While this is a recognised risk factor for suicide in general (World Health Organisation 2014; Ide et al. 2010), this has not been found in other studies of maternal suicide.

There was a high proportion of violent suicide methods in this cohort. This finding contrasts with data on all female suicides in Queensland where violent methods including hanging and jumping from a height are used less commonly (71%, compared with 88% in this study) and non-violent methods such as poisoning by drugs or other means are more common (29%, compared with 12% in this study). This result is consistent with previous research (Cantwell et al. 2011; Appleby 1991; Hogberg et al. 1994; Austin et al. 2007; Esscher et al. 2016).

Less than half of the women who gave birth had records of screening with the EPDS, although it was available more commonly for recent deaths, suggesting that screening is improving. This screening must continue to identify and arrange appropriate prevention strategies for higher-risk women. The EPDS screening tool may not always identify all women at risk, as seen in this cohort where only three women reached the threshold score of thirteen, and this tool should always be used in conjunction with a thorough clinical assessment.

For those women who had a prior mental health diagnosis and no recorded care, there is a question of whether details of their medical history were incomplete. The study also found that a number did not have a previous mental illness. Although much literature exists on the relationship between mental illness and suicide (Joiner et al. 2017; Too et al. 2019), research also indicates that some of those who die by suicide do not have a documented mental health disorder. Factors such as culture, the presence of subclinical disorders, underdiagnosis and service access influence the reporting of information related to the presence of mental illness (Joiner et al. 2017). Additionally, other factors like relationship conflict, domestic violence and low socio-economic status may also influence suicide risk (Brådvik 2018).

The strengths of this study include 14 years of data from two sources, the depth of psychosocial information available from police and coroners’ reports and the inclusion of late maternal deaths. Previous estimates suggest there is a high rate of underreporting of late maternal deaths (Horon and Cheng 2001; Cliffe et al. 2008), which may result in some maternal deaths by suicide not being identified. The likelihood of this in our study is minimal, as Queensland uses rigorous data collection methods involving systematic liaison with the Queensland State Coroner and the Registry of Births, Deaths and Marriages.

The limitations of this study include the small number of deaths and the lack of a specific age-matched comparison group, other than the whole Queensland birthing cohort. Furthermore, psychiatric history mostly came from the deceased’s next of kin or available informants as mental health records were not accessed and were only available if forwarded by the clinician reporting the maternal death. This process may underestimate mental health diagnoses, treatment and medications. Suicide is particularly subject to inaccurate determination (de Leo et al. 2010) as it is not always possible to know the deceased persons’ intent, which may contribute to underestimating suicide mortality. However, these are limitations of all coronial data used for public health surveillance in Australia (Clapperton et al. 2019). Further sample-based research is necessary to confirm the findings from this study.

## Conclusion

Maternal suicide is a rare event. However, it is becoming a leading cause of maternal mortality in Australia. This study indicates that a more concerted effort into perinatal mental health screening and prevention strategies is needed, and these efforts should extend into the later postpartum period. For women with a prior psychiatric history, their ongoing mental health treatment should be addressed at each visit during pregnancy and the postpartum period and should be considered an integral part of their maternity care.

## Supplementary Information

ESM 1 (DOC 78 kb)

ESM 2 (PNG 212 kb)

## Data Availability

Supplied electronically.
