# Supplementary material for: Maternal deaths by suicide in Queensland, Australia, 2004–2017: an analysis of maternal demographic, psychosocial and clinical characteristics
Source: Arch Womens Ment Health. 2021 Jun 22;24(6):1019–25. doi: 10.1007/s00737-021-01107-6 (PMC8585828; doi:10.1007/s00737-021-01107-6)
Supplement: Supplementary file 1 — (DOC 78 kb) [file 737_2021_1107_MOESM1_ESM.doc]

Birth outcome preceding suicide 	
	Frequency	Percent	Valid Percent	Cumulative Percent	
Valid	Abortion	22	33.8	33.8	33.8	
	Pregnancy	6	9.2	9.2	43.1	
	Birth	30	46.2	46.2	89.2	
	Miscarriage	6	9.2	9.2	98.5	
	Stillbirth	1	1.5	1.5	100.0	
	Total	65	100.0	100.0		


Violent or nonviolent method of suicide	
	Frequency	Percent	Valid Percent	Cumulative Percent	
Valid	Violent	57	87.7	87.7	87.7	
	Non-violent	8	12.3	12.3	100.0	
	Total	65	100.0	100.0		
