# Supplementary figures and images for: Maternal deaths by suicide in Queensland, Australia, 2004–2017: an analysis of maternal demographic, psychosocial and clinical characteristics
Source: Arch Womens Ment Health. 2021 Jun 22;24(6):1019–25. doi: 10.1007/s00737-021-01107-6 (PMC8585828; doi:10.1007/s00737-021-01107-6)

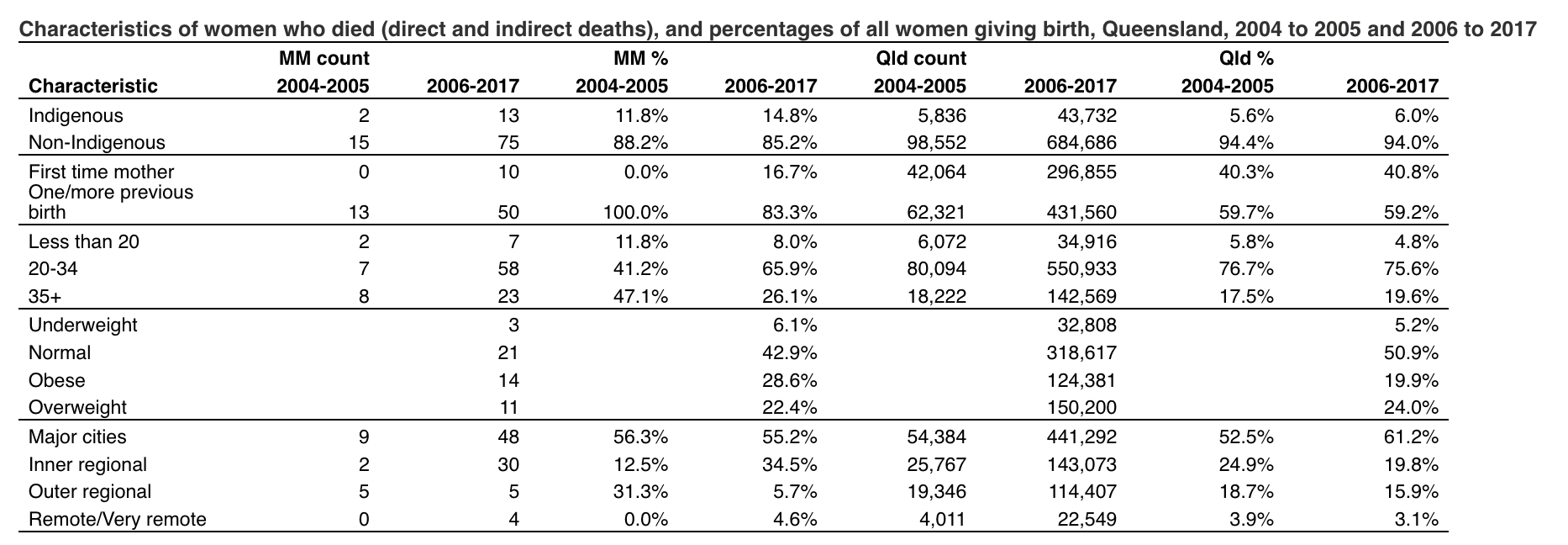

Supplement: Supplementary file 2 — (PNG 212 kb) [file 737_2021_1107_MOESM2_ESM.png]
